# Identification of immune-related hub genes and potential molecular mechanisms involved in COVID-19 via integrated bioinformatics analysis

**DOI:** 10.1038/s41598-024-81803-2

**Published:** 2024-12-02

**Authors:** Rui Zhu, Yaping Zhao, Hui Yin, Linfeng Shu, Yuhang Ma, Yingli Tao

**Affiliations:** 1 School of Pharmacy, Nanjing University of Chinese Medicine, Nanjing, 210023 China; 2 Department of Reproductive Immunology, Tongde Hospital of Zhejiang Province, Hangzhou, 310012, China; 3 Department of Pharmacy, Shaoxing Hospital of Traditional Chinese Medicine Affiliated to Zhejiang Chinese Medical University, Shaoxing, 312000 China; 4 School of Pharmacy, Zhejiang Chinese Medical University, Hangzhou, 310053 China; 5 Animal Science and Technology College, Beijing University of Agriculture, Beijing, 102206 China; 6 School of Traditional Chinese Medicine, Shenyang Pharmaceutical University, Shenyang, 110016 China

**Keywords:** COVID-19, Immune-related genes, Bioinformatics, Mendelian randomization, Immune infiltration, Pathway analysis, Bioinformatics, Gene expression analysis, Genomic analysis, Clinical genetics, Gene expression, Gene regulation, Genetic markers, Genomics, Genetics, Biomarkers, Diseases, Health care, Molecular medicine

## Abstract

COVID-19, caused by the SARS-CoV-2 virus, poses significant health challenges worldwide, particularly due to severe immune-related complications. Understanding the molecular mechanisms and identifying key immune-related genes (IRGs) involved in COVID-19 pathogenesis is critical for developing effective prevention and treatment strategies. This study employed computational tools to analyze biological data (bioinformatics) and a method for inferring causal relationships based on genetic variations, known as Mendelian randomization (MR), to explore the roles of IRGs in COVID-19. We identified differentially expressed genes (DEGs) from datasets available in the Gene Expression Omnibus (GEO), comparing COVID-19 patients with healthy controls. IRGs were sourced from the ImmPort database. We conducted functional enrichment analysis, pathway analysis, and immune infiltration assessments to determine the biological significance of the identified IRGs. A total of 360 common differential IRGs were identified. Among these genes, CD1C, IL1B, and SLP1 have emerged as key IRGs with potential protective effects against COVID-19. Pathway enrichment analysis revealed that CD1C is involved in terpenoid backbone biosynthesis and Th17 cell differentiation, while IL1B is linked to B-cell receptor signaling and the NF-kappa B signaling pathway. Significant correlations were observed between key genes and various immune cells, suggesting that they influence immune cell modulation in COVID-19. This study provides new insights into the immune mechanisms underlying COVID-19, highlighting the crucial role of IRGs in disease progression. These findings suggest that CD1C and IL1B could be potential therapeutic targets. The integrated bioinformatics and MR analysis approach offers a robust framework for further exploring immune responses in COVID-19 patients, as well as for targeted therapy and vaccine development.

**Supplementary Information:**

The online version contains supplementary material available at 10.1038/s41598-024-81803-2.

## Introduction

Severe acute respiratory syndrome coronavirus 2 (SARS-CoV-2) continues to evolve and spread worldwide. As of 18th February 2024, there were 774,699,366 confirmed cases of COVID-19 globally, including 7,033,430 deaths, reported to the World Health Organization (WHO) (https://covid19.who.int/). Due to the highly contagious and variant nature of the virus, ongoing outbreaks of COVID-19 pose significant challenges to the sustainability of global public health systems and medical infrastructure. SARS-CoV-2 utilizes angiotensin-converting enzyme-2 (ACE-2) and transmembrane serine protease-2 (TMPRSS2), which are expressed on type 2 pneumocytes and many other cell types, as receptors to facilitate the fusion of its envelope with the cell membrane and penetration of the cell^1,2^. The main symptoms of COVID-19 include cough, fever, shortness of breath, and pneumonia. The clinical severity ranges from asymptomatic to mild respiratory symptoms and even critical conditions^3^ and may be accompanied by severe complications involving the immune system^4^.

The immune system plays a central role in the pathogenesis of COVID-19. Increasing evidence has shown that immune-related genes are closely associated with the onset and progression of COVID-19 ^5, 6^. An imbalanced immune response during viral invasion is an important immunopathological mechanism in severe disease^7^. In particular, cytokine storms and excessive activation of the immune system are closely associated with the severity of COVID-19 ^8^. Research indicates that a “cytokine storm” may occur in severe COVID-19 and result in a worse prognosis^9,10^. A recent large-scale trial indicated that tocilizumab reduces mortality rates in some groups of hospitalized patients, consistent with previous Mendelian randomization (MR) studies suggesting that IL-6 blockade might be beneficial for inpatient treatment of COVID-19 ^11^. Therefore, considering the close relationship between immune-related genes and the pathogenesis of COVID-19, it is essential to elucidate the biological connections and potential molecular mechanisms between the two to provide new insights into the pathogenesis of COVID-19 and to identify potential therapeutic agents for patients with COVID-19.

Mendelian randomization has been widely employed in assessing causal relationships in the field of disease etiology^12^. By utilizing genetic variations as instrumental variables, potential causal relationships between environmental exposures (such as the levels of certain biomarkers) and outcomes (such as the severity of COVID-19) can be evaluated. Genetic variations are unaffected by confounding factors and randomized during meiosis, MR exhibits a similar ability to extract causal relationships as randomized controlled trials (RCTs)^13^. Therefore, in recent years, when RCTs are impractical or unethical, MR has become an important analytical strategy for handling a range of exposures and outcomes. In addition, since genetic variations precede the onset of disease, MR analysis provides more credible evidence of causality than traditional observational studies, such as cross-sectional studies and case-control studies, by overcoming reverse causality^14^. Previous MR studies have confirmed the causal effects of existing conditions and certain physiological and behavioral variables^15–17^ on the severity of COVID-19, providing many relevant insight. With the advancement of science and technology, bioinformatics has been able to systematically understand biological developmental processes and study disease biomarkers, thus playing a significant role in medical research^18^. The construction of gene interaction networks not only helps to further understand various biological processes from a systemic perspective but can also be widely applied to explore the pathogenesis of diseases. Given the vast amounts of data, classifying differentially expressed genes through enrichment studies is necessary to filter redundant information and identify more valuable functional insights^19^. Commonly used enrichment analyses include GO enrichment analysis, KEGG enrichment analysis, and gene set enrichment analysis (GSEA).

This study utilized bioinformatics methods such as MR to investigate the associations between immune-related genes and COVID-19 and predict their molecular mechanisms of action. First, differential genes were screened based on differential gene expression levels. Datasets from the GEO database for COVID-19 were analyzed to identify differentially expressed genes (DEGs) between the disease and control groups. The immune-related genes (IRGs) were obtained from the ImmPort database, and the DEGs identified in COVID-19 and IRGs were further compared to obtain common DEGs. Subsequently, the study examined the causal relationship between instrumental variables and the outcome. Using eQTL (expression quantitative trait loci) data from common DEGs as exposures and COVID-19 genome-wide association study (GWAS) data as outcomes, the MR method was used to analyze the direct correlation between IRG genes and COVID-19. Next, the IRGs with the highest degree of interaction were identified from the hub genes as potential biomolecules, and we further analyzed the interaction networks between IRGs and transcription factors (TFs), microRNAs (miRNAs), signaling pathways, and other elements. Finally, immune infiltration analysis was employed to uncover molecular regulatory networks and investigate the relationships between IRGs and immune cells. This study provides new insights into the pathophysiological connections and immune mechanisms of IRGs and COVID-19 and provides a foundation for a deeper understanding of the immune response associated with COVID-19.

## Materials and methods

### Study design

**Fig. 1:**
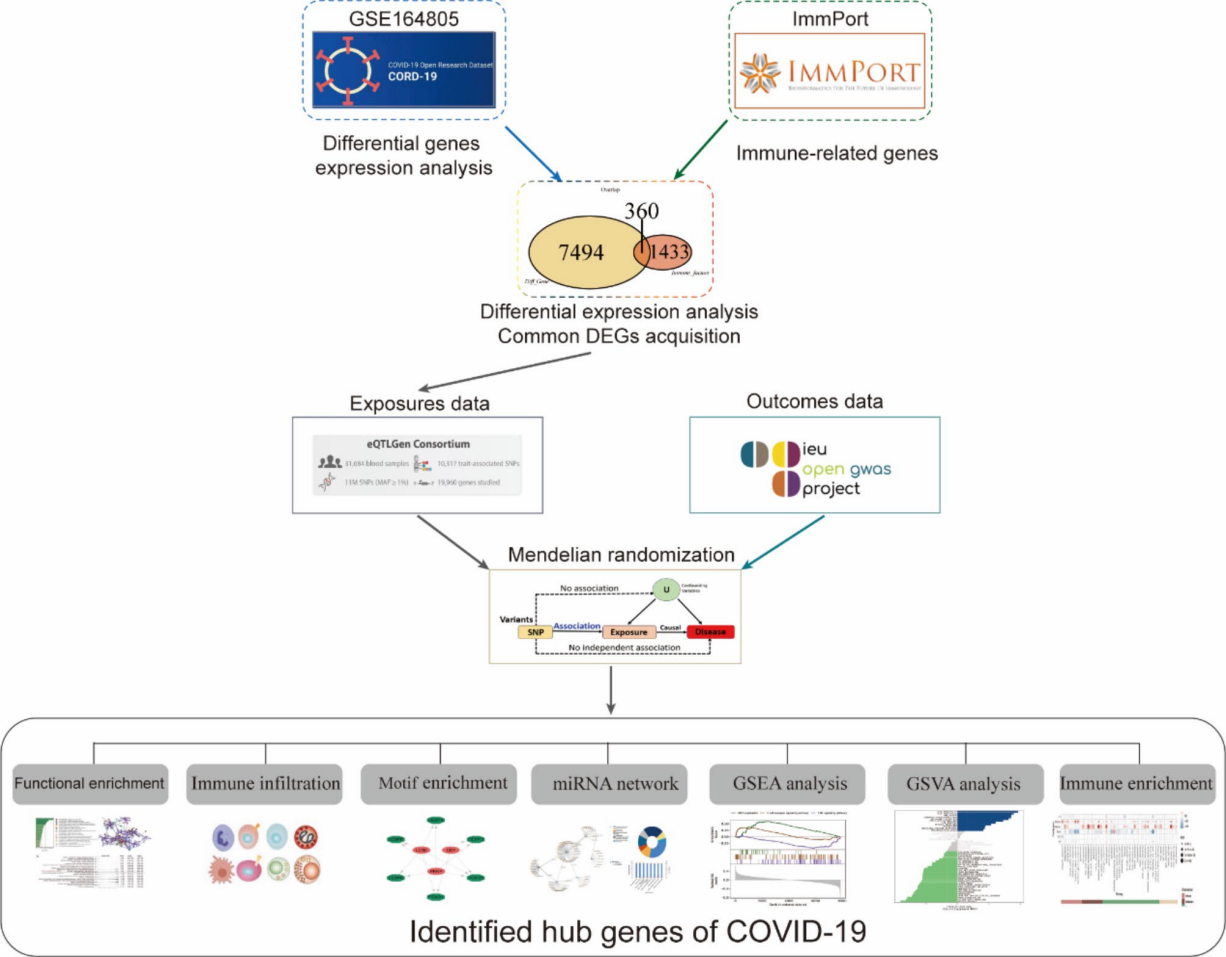
Schematic illustration of the overall general workflow of this study.

A schematic summary of the study design is given in Fig. 1. Initially, differentially expressed genes associated with COVID-19 were screened using the GEO database. These genes were then integrated with immune-related genes from the ImmPort database to identify common immune-related genes (IRGs). Mendelian randomization (MR) analysis was then conducted to investigate potential causal associations between IRGs and COVID-19 risk, utilizing publicly available summary statistics from GWAS. Finally, a series of analyses were performed on the IRGs with the highest degree of interaction, as identified by the MR analysis. These analyses included enrichment analysis, miRNA network analysis, and immune infiltration analysis.

### Data acquisition

(1) GEO database: The COVID-19-related dataset was retrieved from the Gene Expression Omnibus (GEO) database (https://www.ncbi.nlm.nih.gov/geo/)^20^. The training set data were obtained from the GSE164805 Series Matrix File, including samples from 10 COVID-19 patients and 5 healthy control patients. The annotation file utilized was GPL26963. The microarray platform employed was GPL26963 (Agilent-085982 Arraystar human lncRNA V5 microarray)^21^.

(2) Exposures data: Summary statistics from the eQTLGen Consortium (https://www.eqtlgen.org/) were utilized to identify genetic variants associated with blood gene expression levels located within 1000 kb on either side of the coding sequence (in cis). The eQTLGen Consortium dataset includes information on 10,317 single nucleotide polymorphisms (SNPs) associated with traits from 31,684 individuals^22^.

(3) Outcome data: The outcome-related GWAS data used in this study were obtained from the MR database (https://gwas.mrcieu.ac.uk/datasets/ebi-a-GCST011081/). This dataset comprises 9,986 COVID-19 patients and 1,877,672 controls. All GWAS summary data was derived from studies including only individuals of European ancestry to mitigate population stratification bias. The GWAS Catalog contains publications, top associations, and complete summary statistics data, which are currently being mapped to the Genome Assembly and dbSNP Build^23^. All summary data used in this study are publicly available and obtained with the consent and ethical approval of the relevant authorities.

### Differential expression analysis

The Limma package, an R package available from (http://www.bioconductor.org/packages/release/bioc/html/limma.html), is utilized for differential expression analysis of expression profiles, identifying genes that are significantly differentially expressed between various comparative groups^24^. In this study, the R package ‘Limma’ was employed to analyze the molecular mechanisms underlying differences in disease-related data and to identify DEGs between control and disease samples. The parameters for the DEGs screened were set as |log_2_-fold change (FC)| > 1 and adjusted *P* value < 0.05, and volcano plots of the DEGs were created using the ‘ggplot2’ R package. The utilization of a threshold |log_2_-fold change (FC)| > 1 allows for a focus on genes likely to have significant functional implications in the disease process. The adjusted *P*-value threshold of < 0.05 was chosen to control for false discovery rates while ensuring adequate statistical power.

### Functional analysis

To comprehensively explore the functional correlation of these intersecting genes, Metascape (https://metascape.org/gp/index.html#/main/step1) was utilized for the annotation and integration of the DEGs^25^. Gene Ontology (GO)/Kyoto Encyclopedia of Genes and Genomes (KEGG) pathway analysis was performed on specific genes. A minimum overlap ≥ 3 and *p* ≤ 0.01 were used to determine statistical significance.

### Mendelian randomization analysis

The outcome IDs filtered through the MR database were extracted from the GWAS summary data (https://gwas.mrcieu.ac.uk/) to identify relevant causal relationships in eQTLs, and the significance threshold for each gene in the whole locus (*p* < 1 × 10^− 8^)-related SNPs was selected as potential instrumental variables (IVs). The linkage disequilibrium (LD) between SNPs was calculated among SNPs with R^2^ < 0.001 (clumping window size = 10,000 kb), and only *p*^2^ < 5 × 10^− 5^ SNPs were retained. We used four statistical methods, namely, inverse variance weighted (IVW)^26^, MR‒Egger^27^, weighted median^28^, and weighted mode, to assess the reliability of causal relationships and obtain an overall estimate of the impact of the expression of all cis-acting genes and some trans-acting genes on COVID-19 in whole blood. If only one statistical method was applied to a causal relationship, the Wald ratio was used. Sensitivity checks were conducted to validate the robustness of the MR results. Finally, the identified causal relationships were verified and analyzed through leave-one-out validation.

### Immune cell infiltration analysis

Immune cells exhibit specific infiltration and residency patterns, and studying their infiltration status can provide a deeper understanding of their roles and mechanisms in disease pathogenesis. This knowledge can be used to discover new treatment strategies for various diseases^29^. Single-sample gene set enrichment analysis (ssGSEA) is a widely used method to evaluate immune cell types in the microenvironment^30^. This method distinguished 29 human immune cell phenotypes, including T cells, B cells, and NK cells. The ssGSEA algorithm was employed to analyze the proportions of these 29 immune cell types in the GSE164805 dataset, as well as the correlations between immune cells and hub genes, and the inter-correlations among different immune cells.

### Regulatory network analysis of the hub genes

This study utilized the R package “RcisTarget” to predict transcription factors (TFs). All calculations performed by RcisTarget were based on motifs. The normalized enrichment score (NES) for the motifs was determined relative to the total number of motifs in the database. In addition to the motifs annotated by the source data, further annotation files were derived based on motif similarity and the gene sequence. The initial step in evaluating the overexpression of each motif within a gene set involved calculating the area under the curve (AUC) for each motif-gene set pair, based on the recovery curves of the gene sets with respect to motif ordering. The NES for each motif was calculated from the AUC distribution across all motifs in the gene set. The RcisTarget.hg19.motifdb.cisbpont.500 bp was utilized as the gene-motif ranking database.

### miRNA network construction

MicroRNAs (miRNAs) are a subset of small noncoding RNAs that function to modulate gene expression by facilitating mRNA degradation or inhibiting translation. The miRcode database (http://www.mircode.org/) was utilized to search for miRNAs that have the potential to regulate these key genes, and the interactions between these identified miRNAs and the key genes were further investigated. A miRNA network was constructed based on these interactions, followed by an analysis of the network’s structural characteristics and critical nodes. This methodology provides a novel perspective and potential application value for understanding disease mechanisms, identifying disease markers, discovering new drug targets, and improving disease treatment. Consequently, we investigated whether any miRNAs among the immune-related genes (IRGs) regulate the transcription or degradation of these genes.

### Gene set enrichment analysis (GSEA)

GSEA is frequently employed in research that integrates disease classification with biological significance^31^. Accordingly, differential expression analysis was performed on the expression profiles of COVID-19 patients to identify genes differentially expressed between high- and low-expression groups using GSEA (http://www.broadinstitute.org/gsea). The background gene set is an annotated gene set downloaded from the MsigDB database (http://www.gsea-msigdb.org/gsea/msigdb) as an annotated gene set for subtype pathways. Pathway analysis was conducted to assess differential expression between the different subtypes, and gene sets significantly enriched (*p* < 0.05) were selected based on the consistency score.

### Gene set variation analysis (GSVA)

GSVA is a nonparametric and unsupervised method for evaluating transcriptome gene set enrichment^30^. GSVA translates gene-level changes into pathway-level changes by comprehensively scoring gene sets to assess the biological functions of samples. In this study, gene sets were downloaded from the MsigDB database (http://www.gsea-msigdb.org/gsea/msigdb), and the GSVA algorithm was employed to score each gene set comprehensively to evaluate potential changes in biological functions across different samples.

### Statistical analysis

All the statistical analyses were performed using R language (version 4.2), and *p* < 0.05 was considered to indicate statistical significance.

## Results

### Analysis of DEGs between healthy individuals and COVID-19 patients

**Fig. 2:**
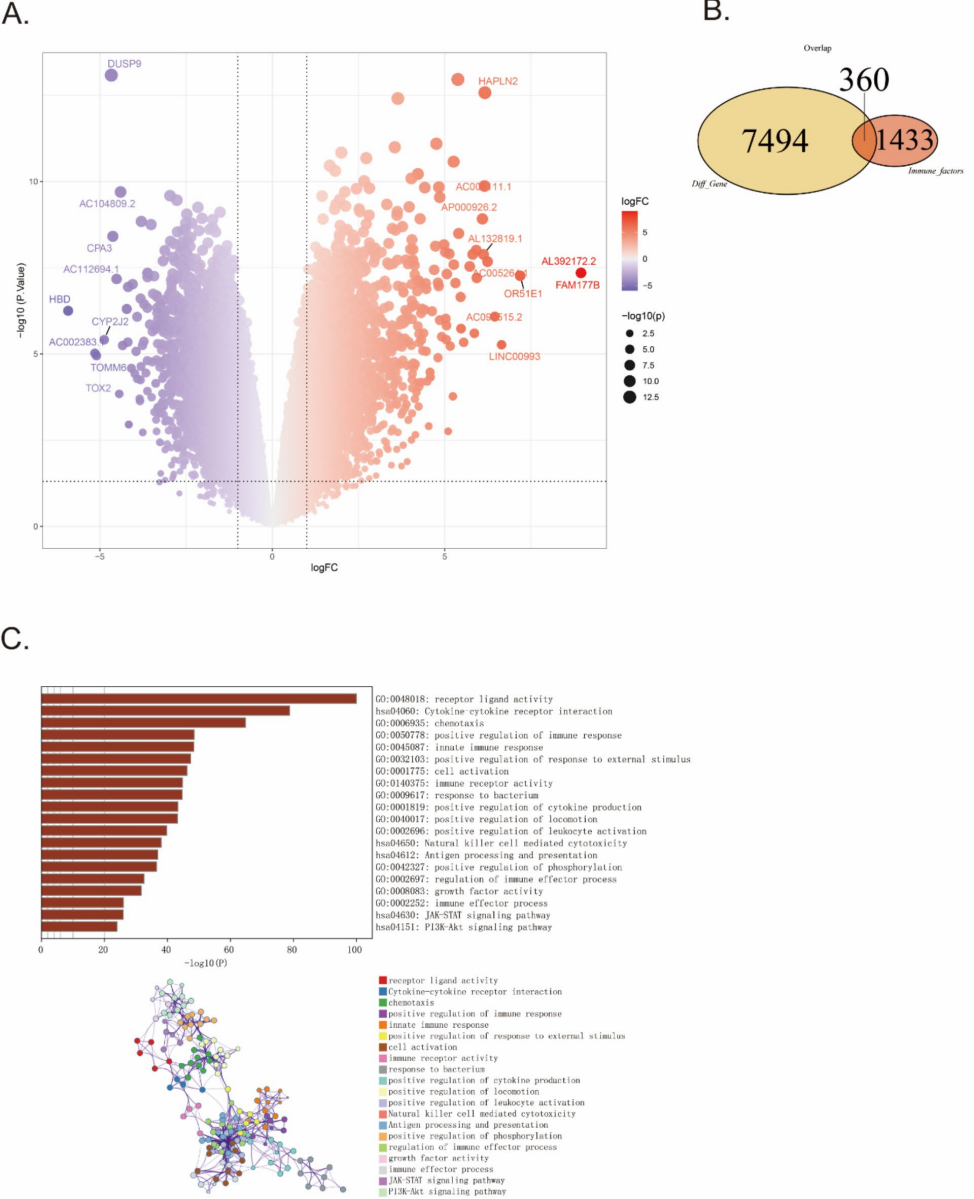
Searching and visualizing differential genes based on GEO and ImmPort databases, and conducting GO/KEGG enrichment analysis. (**A**) The 7854 DEGs volcano plots. The red dots indicate upregulated genes, the purple dots denote downregulated genes, with |log_2_ fold change (FC)|>1 and *P*-value < 0.05. The larger the size of the bubble, the more significant its impact becomes. (**B**) The Venn diagram of the 7854 DEGs and 1433 IRGs genes. A total of 360 DEGs, namely common genes, were obtained. (**C**) The result of GO/KEGG enrichment analysis by metascape database.

Comprehensive gene expression information was obtained from a dataset, GSE164805, comprising 10 COVID-19 patients and 5 healthy controls. We then used the limma package to calculate the DEGs between the two groups of patients. A total of 7,854 DEGs were screened according to parameters including |log_2_-fold change (FC)| > 1 and adjusted *P* value < 0.05, of which 3886 were upregulated genes and 3968 were downregulated genes (Fig. 2A and Supplementary Table 1/2/3). Subsequently, we obtained IRGs from the ImmPort database (https://www.immport.org/home). A total of 1,793 IRGs were obtained from ImmPort and intersected with the 7,854 DEGs to obtain 360 common differential IRGs, as shown in the Venn diagram (Fig. 2B). GO and KEGG enrichment analyses were performed on the 360 common IRGs to examine their biological roles and signaling pathways. It revealed significant enrichment of the IRGs in various biological processes (BP), including chemotaxis (GO:0006935), positive regulation of immune response (GO:0050778), innate immune response (GO:0045087), positive regulation of response to external stimulus (GO:0032103), response to bacterium (GO:0009617), positive regulation of cytokine production (GO:0001819), positive regulation of locomotion (GO:0040017), positive regulation of leukocyte activation (GO:0002696), positive regulation of phosphorylation (GO:0042327), regulation of immune effector process (GO:0002697), immune effector process (GO:0002252). The IRGs were also involved in different molecular functions (MF), including receptor ligand activity (GO:0048018), cell activation (GO:0001775), immune receptor activity (GO:0140375), growth factor activity (GO:0008083). Furthermore, multiple immune-related pathways were also enriched, including Cytokine-cytokine receptor interaction (hsa04060), Natural killer cell mediated cytotoxicity (hsa04650), Antigen processing and presentation (hsa04612), JAK-STAT signaling pathway (hsa04630) and PI3K-Akt signaling pathway (hsa04151). All results were visualized using bar plots and network diagram, which manifested that 360 common IRGs might be involved in immune-related functions and pathways (Fig. 2C). Research has shown that targeting the JAK-STAT signaling pathway can control cytokine release in patients with COVID-19 ^32^, and targeting the PI3K/Akt/mTOR pathway is a potential strategy for treating COVID-19 ^33^. Currently developed vaccines rely on the natural spike protein (S) of SARS-CoV-2 to induce strong neutralizing antibodies; however, the way this key antigen is presented to the immune system varies significantly among different types of vaccines^34^. The JAK-STAT and PI3K-Akt signaling pathways, as well as the presentation of COVID-19 vaccine antigens involved in these studies, align with the results of our GO/KEGG functional enrichment analysis of the 360 IRGs, indicating a strong correlation between the biological functions of IRGs and the pathogenesis of COVID-19. This will aid in guiding COVID-19 treatment strategies and the development of related vaccines.

### Causal effects of IRGs on COVID-19

**Fig. 3:**
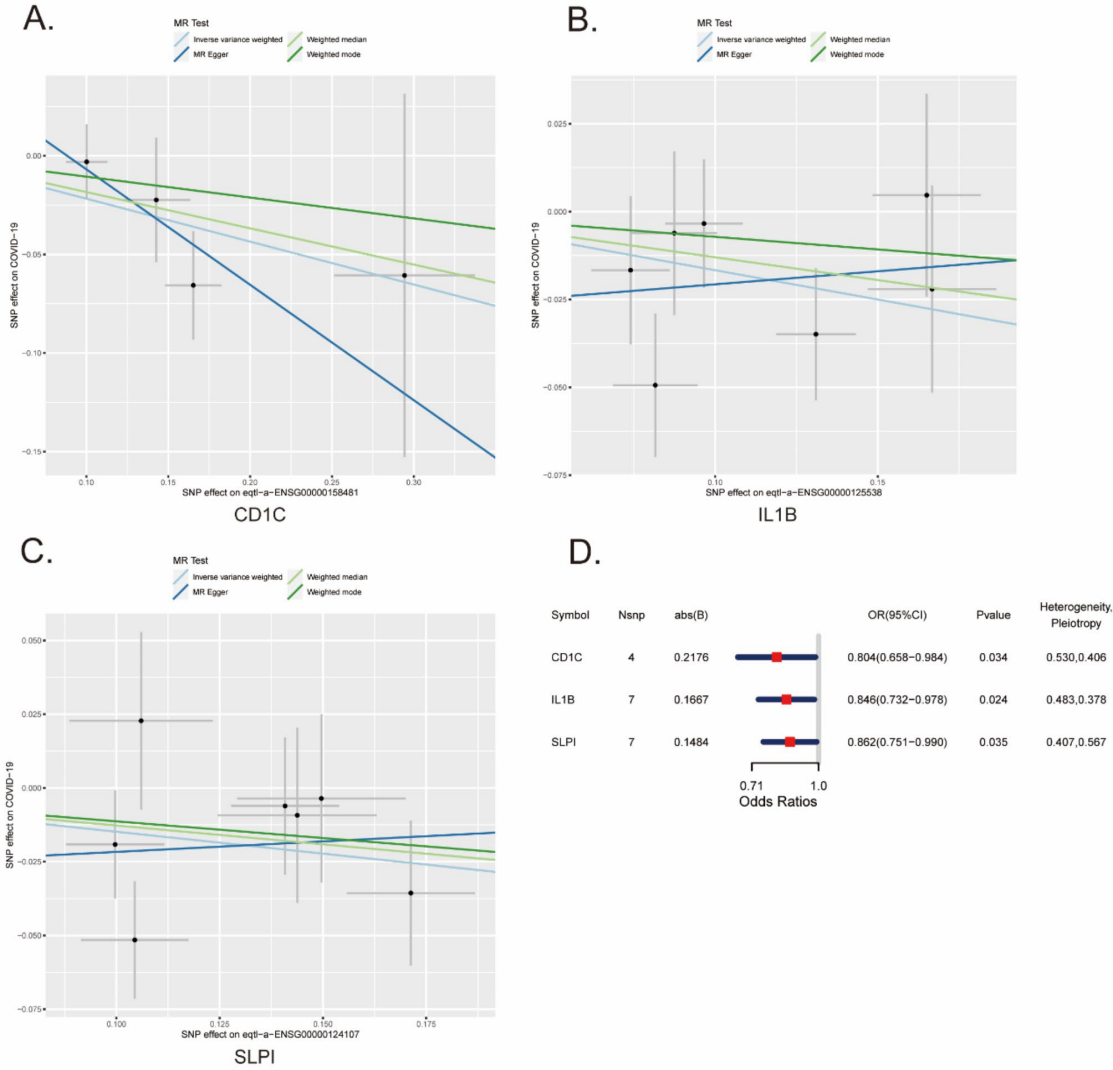
Mendelian randomization analysis. (**A**) Scatter plots of the causal effect of CD1C on COVID-19 in the MR analysis. (**B**) Scatter plots of the causal effect of IL1B on COVID-19 in the MR analysis. (**C**) Scatter plots of the causal effect of SLPI on COVID-19 in the MR analysis. (**D**) Forest plot of MR estimates for the association between IRGs and COVID-19. Lines represent the beta effect between IRGs and COVID-19 using inverse variance weighted (light blue), MR-Egger (dark blue), weighted median (green), and weighted mode (dark green). Points indicate the betas of each instrument with IRGs and COVID-19. Vertical and horizontal lines centered at each point show 95% confidence intervals for the associations of each instrument with IRGs and COVID-19. OR: odds ratio; CI: confidence interval. Points indicate the odds ratio per standard deviation increment increase in COVID-19. Error bars indicate 95% confidence intervals.

**Fig. 4:**
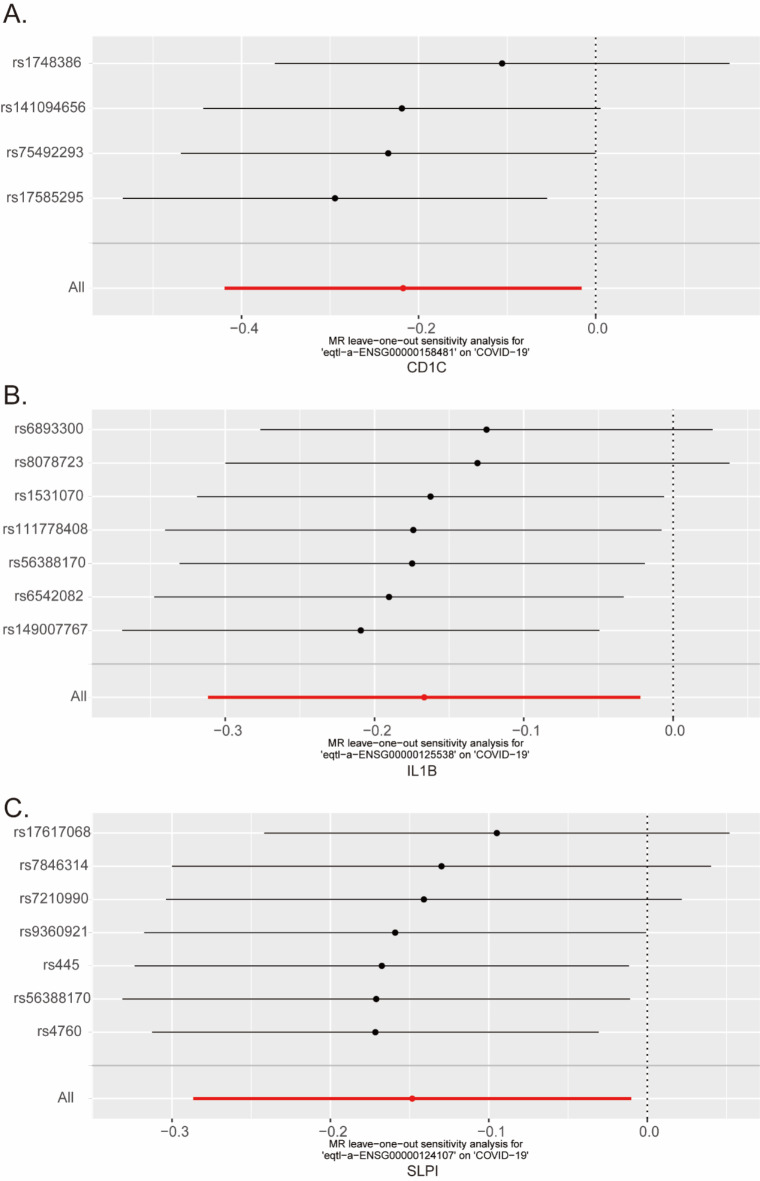
Leave-one-out sensitivity analysis. (**A**) Leave-one-out sensitivity analysis of the effect of individual SNPs on the association between CD1C and COVID-19. (**B**) Leave-one-out sensitivity analysis of the effect of individual SNPs on the association between IL1B and COVID-19. (**A**) Leave-one-out sensitivity analysis of the effect of individual SNPs on the association between SLPI and COVID-19.

To further identify the key genes affecting COVID-19, we employed the MR method based on the causal relationship between instrumental variables and the outcome for our analysis. We used the eQTLs of the 360 IRGs as exposure factors and the outcome factor ID (ebi-a-GCST011081) was obtained through the summary statistics of 1,887,658 COVID-19-related samples (controls: 1,877,672; cases: 9,986). Using the functions extract_instruments and extract_outcome_data, we extracted exposure and outcome SNPs, we obtained 206 pairs of closely related genes between exposure and outcome (Supplementary Table 4). Furthermore, the causal relationships of 3 pairs of genes corresponding to the positive eQTL outcome were obtained by MR analysis (IVW, *P* value < 0.05), and the corresponding genes were CD1C, IL1B, and SLPI. The presence of CD1C (IVW OR = 0.804, 95% CI: 0.658 − 0.984, *p* = 0.034), IL1B (IVW OR = 0.846; 95% CI: 0.732–0.978; *p* = 0.024), and SLPI (IVW OR = 0.862; 95% CI: 0.751–0.990; *p* = 0.035) may be associated with a lower risk of COVID-19 (Fig. 3). To assess the reliability of the causal relationships identified for the three genes, a leave-one-out sensitivity analysis was performed to determine the reliability of the MR data. The results indicate that excluding any single SNP does not significantly affect the overall results, suggesting that these causal relationships are robust (Fig. 4). Therefore, these three genes composed the candidate gene set for subsequent analysis, and the genes were in the following order: CD1C, IL1B, and SLPI.

### Expression and predictive analysis of IRG genes in COVID-19 patients

**Fig. 5:**
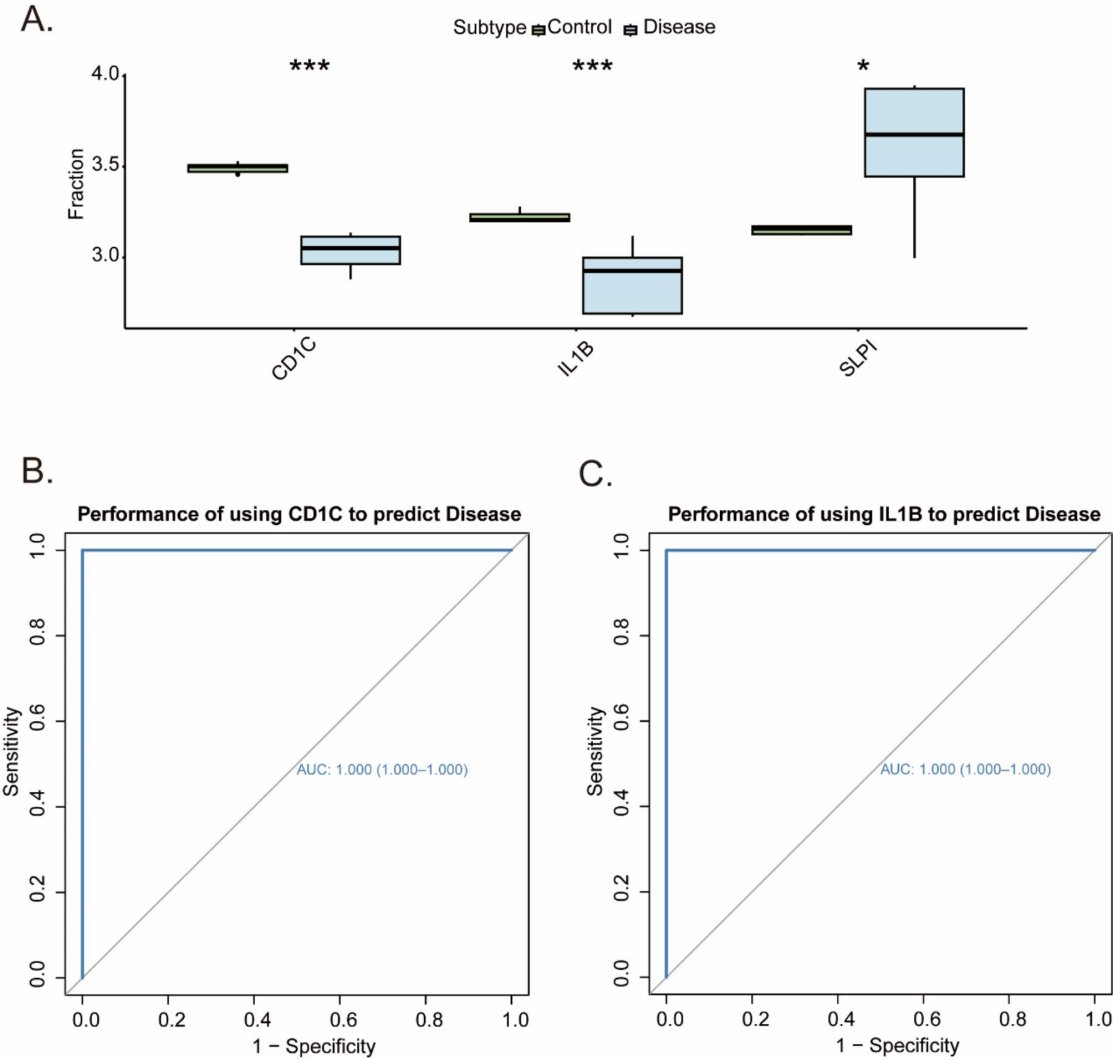
Differential expression analysis of IRGs in the disease and control groups of COVID-19 data. (**A**) The expression differences of IRGs in the disease and control groups of COVID-19 data by box plot depicting. (**B**) The performance of ROC curves about CD1C gene. (**C**) The performance of ROC curves about IL1B gene.

Analyzing the expression of genes in disease and control groups of COVID-19 patients is crucial for understanding the molecular mechanisms underlying the disease and identifying potential therapeutic targets. Therefore, we further analyzed IRG expression in the disease and control groups of COVID-19 patients. The results showed significant differences in the expression of three genes in both patient groups. Moreover, the trends observed in the MR analysis were consistent with those of the CD1C and IL1B genes (Fig. 5A). Therefore, we identified CD1C and IL1B as key genes for further analysis. Additionally, we evaluated the predictive performance of these key genes using receiver operating characteristic (ROC) curves to assess diagnostic efficacy. A higher AUC indicates better predictive performance^35^. The AUC values for the two key genes were as follows: CD1C-AUC of 1.000 (95% CI: 1.000–1.000) and IL1B-AUC of 1.000 (95% CI: 1.000–1.000) (Fig. 5B and C), suggesting that both key genes can effectively predict the onset and progression of the disease.

### Analysis of the correlation among IRGs, immune cells, and COVID-19 through multiomics studies

**Fig. 6:**
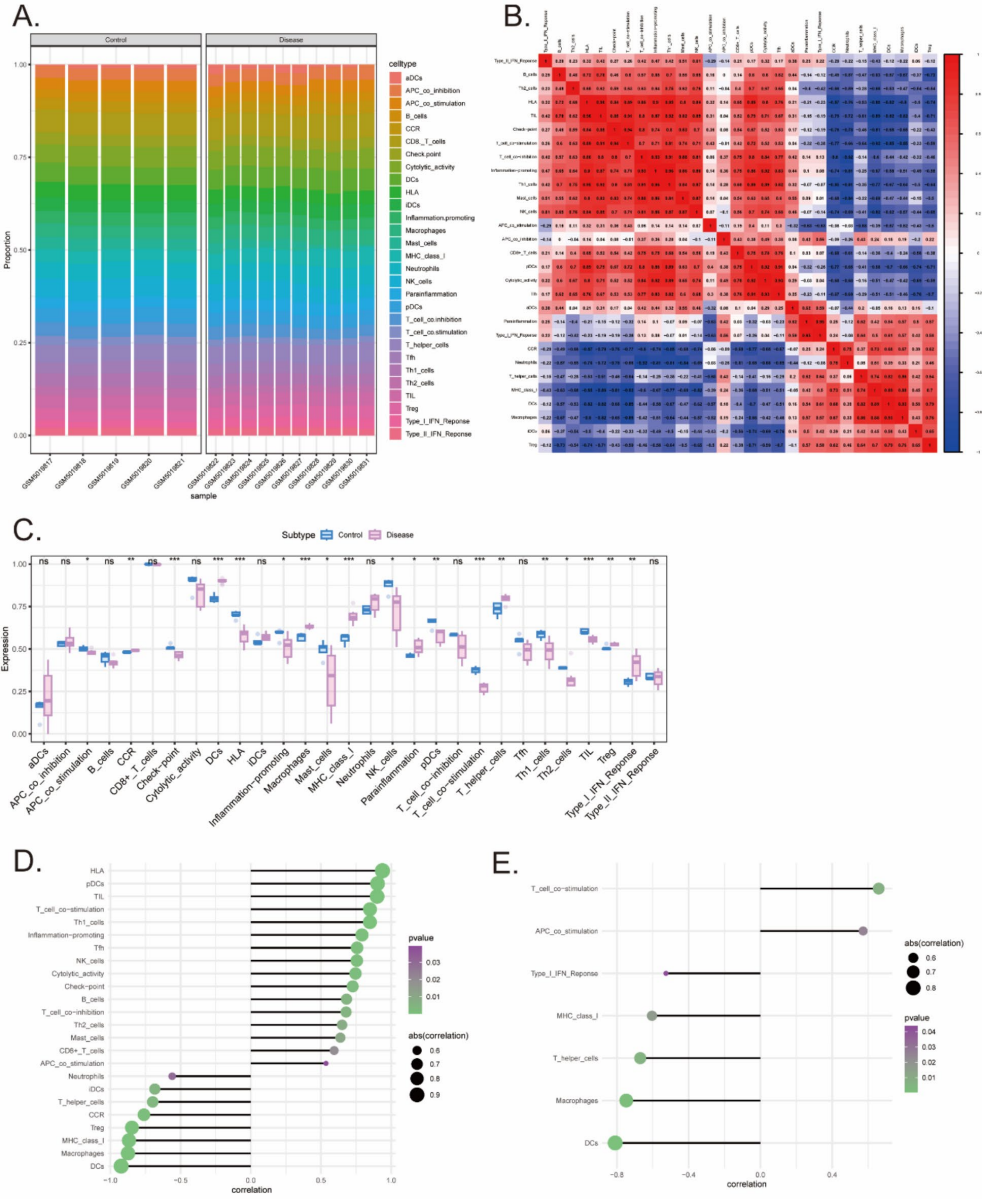
Immune cell infiltration analysis. (**A**) Stacked bar chart of percentages of 29 immune cells in the disease and control groups of COVID-19. (**B**) Pearson correlation heat map among 29 types of immune cells. The size of the colored squares represents the strength of the correlation. Red represents a positive correlation, blue represents a negative correlation. The darker the color, the stronger the correlation. (**C**) The differences in abundance of 29 immune cell types between the disease and control groups of COVID-19, illustrated by box plots. Blue represents the control group, while purple represents the COVID-19 group. (**D**) Visual representation of relationships among CD1C gene and immune cells by Lollipop chart. (**E**) Visual representation of relationships among IL1B gene and immune cells by Lollipop chart.

The common immune cells include neutrophils, eosinophils, basophils, lymphocytes, and macrophages. The role of immune cells in the body’s resistance to infection and removal of foreign bodies is well-recognized. We further explored the relationships between two key genes identified in the COVID-19 GWAS dataset and immune infiltration, aiming to elucidate the potential molecular mechanisms through which these key genes influence the progression of COVID-19. Finally, the proportions of immune cells in each patient (Fig. 6A) and the correlations between different immune cells (Fig. 6B) are presented in various forms. In addition, the results of the present study indicated that compared with control patients, patients in the disease group had significantly greater numbers of immune cells, such as human leukocyte antigen (HLA), dendritic cells (DCs), macrophages, and major histocompatibility complex (MHC) class I (Fig. 6C). This study also explored the relationship between the CD1C and IL1B genes and immune cells, with the results indicating a strong correlation between CD1C and IL1B genes and immune cells (Fig. 6D and E). For instance, CD1C showed a significant positive correlation with HLA and a significant negative correlation with DCs. Similarly, IL1B exhibited a significant positive correlation with T-cell costimulation and a significant negative correlation with DCs. These findings imply that CD1C and IL1B may influence the onset and progression of COVID-19 through their impact on immune cells, further suggesting that modulating immune cell infiltration may be key to managing disease severity.

### Regulatory network analysis of the interactions among transcription factors, mRNA‒miRNA interaction pairs, and IRGs

**Fig. 7:**
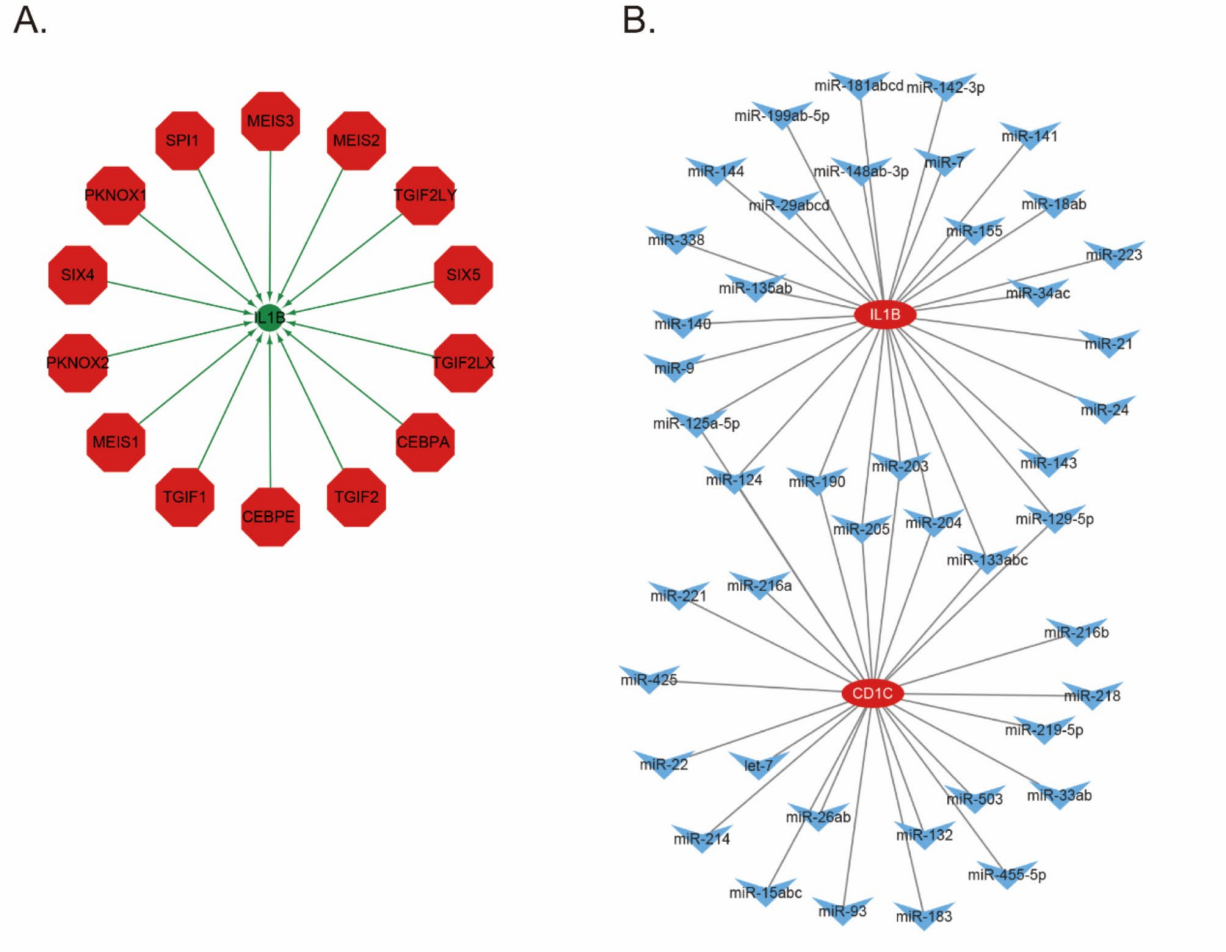
Regulatory network analysis of the interactions among transcription factors, mRNA-miRNA interaction pairs, and IRGs. (**A**) Regulatory network between IRGs genes and transcription factors, with IRGs represented in green and transcription factors represented in red. (**B**) Regulatory network between IRGs genes and mRNA-miRNA interaction pairs, with IRGs represented in red and miRNA represented in blue.

Subsequently, two key genes were selected for the gene set used in this analysis, and they were found to be regulated by common mechanisms such as transcription factors. Therefore, we performed enrichment analysis for transcription factors. The enrichment analysis involved three steps: (1) motif enrichment analysis with cumulative recovery curves, (2) motif-TF annotation, and (3) selection of significant genes. The results indicated that the motif cisbp__M4410 had the highest standardized enrichment score (NES: 6.18). This study revealed all the enriched motifs and their corresponding transcription factors for the two key genes analyzed (Fig. 7A and Supplementary Table 5). Additionally, we performed reverse prediction on the two key genes using the miRcode (http://www.mircode.org/index.php) database, resulting in 44 miRNAs and a total of 52 mRNA‒miRNA interaction pairs. We visualized these interactions using Cytoscape (Fig. 7B).

### Enrichment analysis of the involvement of IRGs in COVID-19-related signaling pathways

**Fig. 8:**
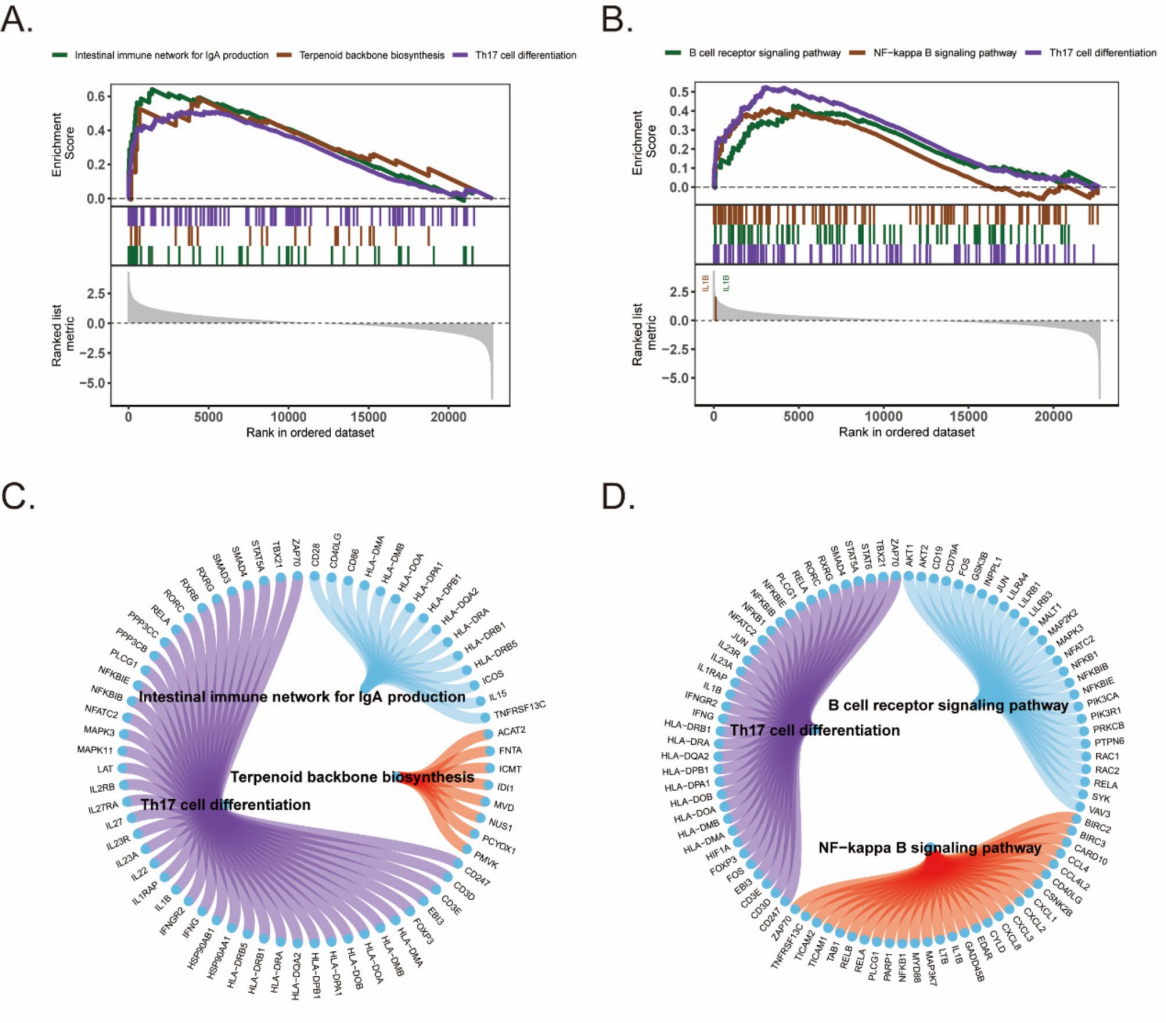
GSEA analysis of IRGs genes. (**A**) Visualizing the enrichment results of signaling pathways associated with CD1C genes by gseaplot using GSEA analysis. (**B**) Visualizing the enrichment results of signaling pathways associated with IL1B genes by gseaplot using GSEA analysis. (**C**) Visualizing the enrichment results of genes involved in signaling pathways associated with CD1C by a petalplot. (**D**) Visualizing the enrichment results of genes involved in signaling pathways associated with IL1B by a petalplot.

**Fig. 9:**
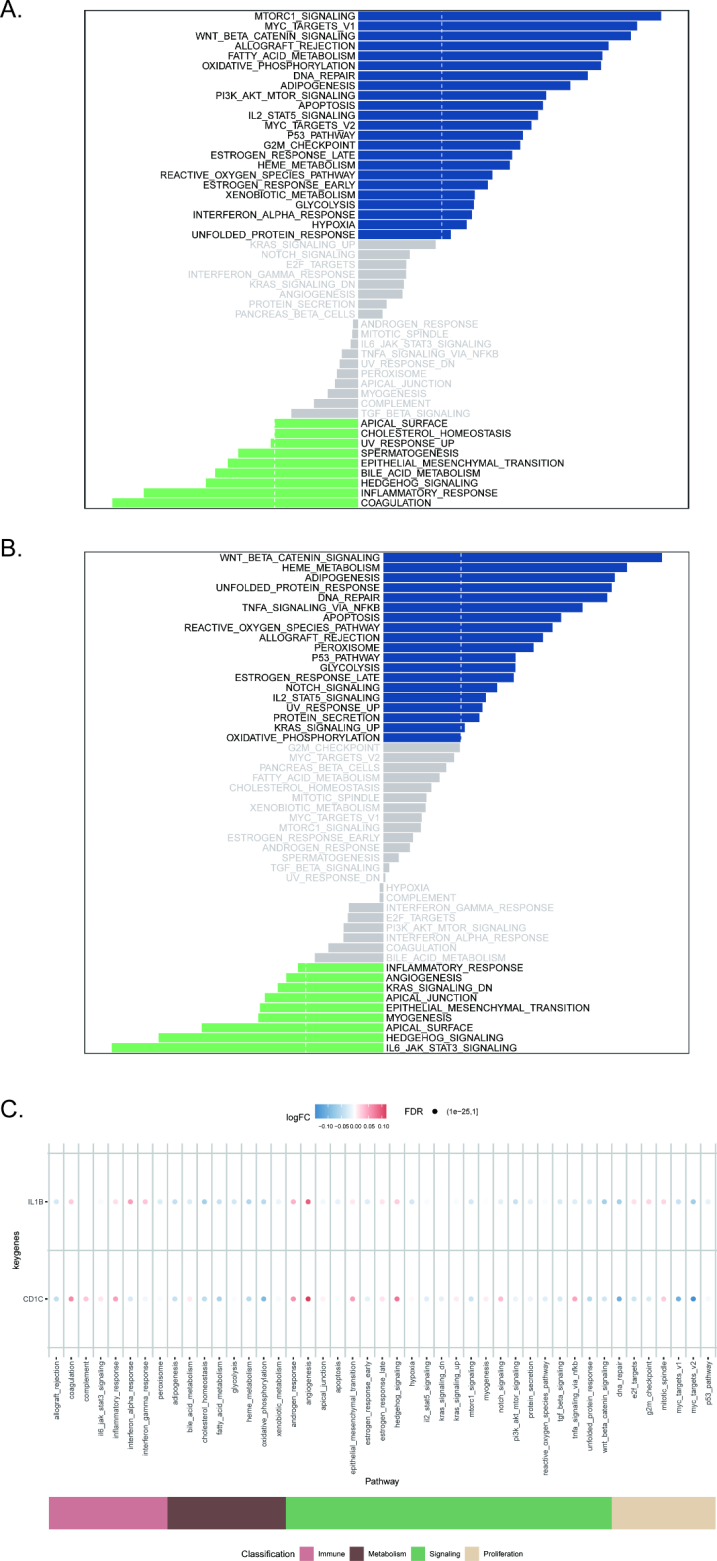
Analysis of the correlation between CD1C or IL1B expression and immune metabolism signaling pathways. (**A**, **B**). GSVA analysis of the correlation between CD1C or IL1B and immune metabolism signaling pathways presented through a diverging bar plot. Blue indicates signaling pathways associated with high expression of genes, while green indicates signaling pathways associated with low expression of genes. The background gene set is Hallmark. (**C**) Visualizing the enrichment results of immunity, metabolism, and pathways associated with CD1C or IL1B gene using ssGSEA analysis.

We further explored the correlation between the two key genes and signaling pathways to investigate the potential molecular mechanisms underlying the association between the key genes and COVID-19. The GSEA results showed that CD1C was enriched in pathways such as terpenoid backbone biosynthesis and Th17 cell differentiation (Fig. 8A). IL1B was enriched in pathways such as the B-cell receptor signaling pathway and the NF-kappa B signaling pathway (Fig. 8B). Using the ccgraph package, a petal plot of the genes involved in the pathway can be drawn simultaneously (Fig. 8C and D). The GSVA results indicated that high expression of CD1C was associated with enrichment in signaling pathways such as the mtorc1 signaling and IL2-Stat5 signaling pathways (Fig. 9A). High expression of IL1B is associated with enrichment of signaling pathways such as the Wnt-β-Catenin Signaling, p53 pathway (Fig. 9B). Finally, we quantified immune and metabolic pathways using ssGSEA and analyzed the correlations between the two key genes and immune and metabolic pathways (Fig. 9C).

## Discussion

Immune system disorders are suspected to play a role in severe COVID-19 risk^36,37^. Increasing evidence suggests a close association between COVID-19 and the immune response, with variations observed among different populations^38^. After SARS-CoV-2 infects the human body, it triggers an inflammatory immune response, during which white blood cells from the lymph nodes, such as helper T cells and cytotoxic T cells, infiltrate the site of infection to eliminate virus-infected cells^39^. In patients with more severe illness, SARS-CoV-2 induces abnormal host immune responses^40^. COVID-19 is a real-time global pandemic, and this viral infection may also trigger other immune-related diseases. Consequently, numerous studies have explored the correlation between COVID-19 and immune cells^4,41,42^. Currently, research on the correlation between COVID-19 and immune cells has focused mainly on basic research and vaccine studies, with relatively few studies utilizing bioinformatics to explore this correlation, and the underlying mechanisms are still lacking. Therefore, our study aimed to reveal the correlation and potential mechanisms between immune-related genes and COVID-19 from a molecular regulatory perspective through bioinformatics analysis and MR analysis to provide a theoretical basis for the treatment of COVID-19.

Enrichment analysis of pathways and functions helps us to understand the regulatory effects and specific mechanisms of genes in the body. First, this study downloaded a COVID-19 dataset from the GEO database and obtained 7,854 DEGs through differential expression analysis. Subsequently, 1,793 IRGs were obtained from the ImmPort database, and the intersection with the 7,854 DEGs yielded 360 common differential IRGs, which were then subjected to functional enrichment analysis. The enrichment analysis results indicated that these differentially expressed IRGs were enriched mainly in pathways and functions related to immunity and inflammation, such as the cytokine‒cytokine receptor interaction signaling pathway, the positive regulation of the immune response signaling pathway, the JAK-STAT signaling pathway, and the PI3K-Akt signaling pathway. Differentially expressed IRGs play crucial roles in the pathogenesis of COVID-19. There is substantial evidence indicating that IRGs are associated with COVID-19 ^43,44^. For example, studies have shown that increased expression of the IRF9, IFI6, OAS1, CCL5, and TGFB1 genes was detected in individuals infected with the SARS-CoV-2 wild-type virus, possibly due to evasion of the immune response to viral variants and/or vaccination^44^. Given these findings, we will continue to identify the most relevant genes from the 360 IRGs for further research related to COVID-19.

Previous observational studies have frequently shown changes in immune-related genes in patients with severe COVID-19 compared to healthy controls or mild COVID-19 patients^45,46^. However, observational studies can be influenced by confounding factors, making it difficult to distinguish between symptoms and causes. Therefore, in this MR analysis, we used 360 differential immune-related gene (IRG) eQTLs as exposure factors and COVID-19 (ebi-a-GCST011081) as the outcome. By employing Mendelian randomization analysis, we identified the 3 most relevant genes: CD1C, IL1B, and SLP1. These 3 genes may act as protective factors in COVID-19 patients. The leave-one-out analysis results indicate that excluding any single SNP does not significantly alter the overall error bar, suggesting that the three selected pairs of causal relationships are robust. Building on these findings, we further analyzed the expression of IRGs in the disease and control groups of COVID-19 patients. The significant downregulation of CD1C and IL1B in the COVID-19 patient group, consistent with the MR analysis results, underscores their potential role as protective genes.

CD1C is a member of the CD1 gene family, which is located on chromosome 1 outside the MHC region in humans^47^. The CD1C protein is classified as a member of the MHC class I-like protein family and is prominently expressed on the surface of dendritic cells^48,49^. CD1C plays a pivotal role in the pathogenesis of inflammatory diseases. Previous research has established a correlation between the abundance of CD1C + dendritic cells and conditions such as Langerhans cell histiocytosis (LCH), COVID-19, and systemic lupus erythematosus (SLE)^50–52^. CD1C’s role in dendritic cell function suggests its importance in modulating the immune response during COVID-19. This study found a more pronounced reduction in CD1C levels among patients with severe COVID-19. CD1C + conventional dendritic cells showed a preference for migrating from the blood to the lungs in these patients^50^, which is consistent with our study that CD1C is downregulated in COVID-19 patients and functions as a protective gene. IL-1B, a member of the IL-1 family, is a proinflammatory cytokine primarily synthesized by tissue macrophages^53,54^ and is potentially secreted from various other cell types, including neutrophils and epithelial cells^55–59^. The expression of the IL1B gene in COVID-19 patients is closely associated with the TLR4 signaling pathway^60^. Projection of RNA-seq data on SARS-CoV-2–infected human bronchial epithelial cells showed that several inflammatory cytokines and chemokines, such as IL-1B, were downregulated in the intestines of patients with COVID-19 ^61^. In our investigation, we also observed downregulation of IL1B in individuals affected by COVID-19, consistent with prior findings documented in the scientific literature. In addition, we investigated the predictive performance of the CD1C and IL1B genes using ROC curves validated by diagnostic efficacy. A higher AUC indicates superior predictive performance. Overall, these findings underscore the potential of CD1C and IL1B as biomarkers for predicting the onset and progression of COVID-19, which could inform therapeutic strategies.

In this study, we further explored the relationship between CD1C and IL1B genes discovered in the COVID-19 GWAS dataset and immune infiltration. These findings indicated a heightened presence of dendritic cells, macrophages, MHC-I molecules, T helper cells, and IFN-γ, as well as a diminished presence of Th1, Th2, and HLA in individuals with COVID-19. Other studies have also demonstrated that respiratory dendritic cells play a central role in the pathogenesis of SARS-CoV-2 ^62^. In addition, Rogan presented a dataset that supports a testable model in which alveolar macrophages containing SARS-CoV-2 form positive feedback loops with IFNγ-secreting T cells to promote alveolitis in patients with severe COVID-19 ^63^. Fatoba et al. demonstrated that CD4 + T-cell epitopes are capable of inducing the production of the cytokines IFN-γ and IL-4, while the highly conserved MHC-I and MHC-II epitopes of both CD8 + and CD4 + T cells can bind to TLR3 receptors^64^. The predominant subpopulations of T helper lymphocytes (Th) in critically ill COVID-19 patients are Th1, Th2, Th17 (without their main characteristics) and regulatory T cells (Tregs). Some CD8 + T lymphocyte markers, such as human leukocyte antigen (HLA-DR) and programmed cell death protein 1 (PD-1), are associated with disease severity^65^. These findings establish a foundation for the experimental development of an effective vaccine against SARS-CoV-2, as well as potential therapeutic interventions. Recent research has demonstrated a close correlation between human leukocyte antigen (HLA) alleles and asymptomatic SARS-CoV-2 infection^66^. Furthermore, plasmacytoid dendritic cells (pDCs) are susceptible to SARS-CoV-2 infection and can produce interferons, thereby inducing epigenetic modifications in macrophages located in the vicinity of the lungs in infected individuals^67^. During the acute phase of SARS-CoV-2 infection, there is a widespread decrease in immune cell populations, including T cells, NK cells, monocytes, and dendritic cells (DCs)^68^. Hence, by integrating prior findings with our own experimental data on immune cell infiltration, it is hypothesized that the CD1C and IL1B genes may modulate the onset and progression of SARS-CoV-2 through their impact on immune cell populations such as HLA, pDCs, T cells, and NK cells.

To determine the transcriptional regulation of COVID-19 by common IRGs, we utilized web tools to investigate the interactions among TFs, miRNAs, and genes. Our results revealed a regulatory relationship between TFs (SPI1, MEIS3, MEIS2, TGIF2LY, SIX5, TGIF2LX, CEBPA, TGIF2, CEBPE, TGIF1, MEIS1, PKNOX2, SIX4, and PKNOX2) and miRNAs (miR-125a-5p, miR-124, miR-190, miR-205, miR-203, miR-204, miR-133abc, miR-129-5p. etc.) and genes (CD1C, IL1B) that may play important roles in COVID-19. Pulugulla et al. elucidated the structural basis of the C/EBP-Spi1 interaction essential for IL1B gene transcription using a combination of computational and experimental approaches^69^. Previous studies revealed that the MEIS transcription factor plays a crucial role in regulating mouse limb development patterns^70^. Additionally, the SIX5 transcription factor is involved in regulating plasma cell-specific activities and is closely linked to plasma cell maturation^71^. Mice with mutations in the CEBPA gene exhibit selective blockage of granulocyte development at an early stage, which is a characteristic hallmark of acute myelogenous leukemia (AML)^72^. Recently, research on miRNAs has revealed a strong correlation between miRNAs and the process of postinfarction heart repair, as well as atopic dermatitis and other immune-related diseases^73,74^. Although many previous studies and the present study have indicated the potential therapeutic impact of these TFs and miRNAs, additional experimental validation is necessary to confirm their validity and authenticity.

A gene-signaling pathway relationship network was established to investigate the potential molecular mechanisms underlying the association between the IRGs and COVID-19, and these results can inspire us to develop novel therapeutic agents to treat COVID-19 through the occurrence, development, and treatment of these diseases. The differentiation of Th17 cells, a subset of CD4 + T cells that play an important role in combating extracellular microorganisms and autoimmune diseases in vivo, requires antigen presentation and costimulation, as well as the activation of antigen-presenting cells (APCs) to produce cytokines. Our GSEA results showed that Th17 cell differentiation was affected by either the CD1C or IL1B gene. Research also indicates that Th17 differentiation is closely related to COVID-19 ^75^, so it can be inferred that CD1C and IL1B may influence the occurrence and development of COVID-19 through the Th17 differentiation pathway. Additionally, we identified significant enrichment of the intestinal immune network for IgA production and terpenoid backbone biosynthesis in the CD1C gene, while the IL1B gene exhibited significant enrichment of the B-cell receptor signaling pathway and NF-kappa B signaling pathway. This finding suggested that the functions of CD1C and IL1B are mainly involved in the activation of immune cells. The GSVA results revealed a positive correlation between CD1C and IL1B and Wnt-β-Catenin Signaling, p53 pathway, and IL-12-Stat5 Signaling, as well as a negative correlation with inflammatory response, apical surface, and epithelial mesenchymal transition. These findings suggest that CD1C and IL1B may act as protective factors in COVID-19 by participating in various immune signaling pathways. The ssGSEA method was employed to assess the association between CD1C and IL1B genes and immunity in terms of immune response, metabolism, signaling pathways, and proliferation. The findings suggest a positive correlation between the IL1B and CD1C genes and processes such as vascular genesis, hedgehog signaling, androgen response, and epithelial mesenchymal transition, while indicating a negative correlation with allograft rejection, adipogenesis, cholesterol homeostasis, and oxidative phosphorylation.

## Conclusion

In recent years, extensive research and discussion have focused on the intricate relationships between immune-related genes and the onset and progression of COVID-19. However, there is a dearth of exploration and investigations into the specific immune genes closely associated with COVID-19 and their underlying mechanisms. In this study, we utilized the eQTL (expression quantitative trait locus) data of 360 differentially expressed immune-related genes as exposures and COVID-19 GWAS data as outcomes. Through the application of Mendelian randomization methodology, two immune genes, CD1C and IL1B, have been identified as exhibiting the closest association with COVID-19. Furthermore, these two genes confer a protective effect against COVID-19. Subsequently, this study performed enrichment analysis and constructed various interaction networks to elucidate the associations among CD1C, IL1B, immune infiltration, transcription factors, miRNAs, and signaling pathways from multiple perspectives, aiming to further investigate the potential molecular mechanisms underlying the impact of CD1C and IL1B on COVID-19. Building upon the aforementioned research foundation, future investigations will be undertaken to delineate the functions and mechanisms of the CD1C and IL1B genes to offer novel insights for identifying potential biomarkers and exploring suitable therapeutic strategies for COVID-19.

## Electronic supplementary material

Below is the link to the electronic supplementary material.

Supplementary Material 1

Supplementary Material 2

Supplementary Material 3

Supplementary Material 4

Supplementary Material 5

## Data Availability

The datasets generated during the current study are available from the corresponding author on reasonable request.

## Abbreviations

SARS-CoV-2: Revere Acute Respiratory Syndrome Coronavirus 2
ACE-2: Angiotensin Converting Enzyme-2
TMPRSS2: Transmembrane Serine Protease-2
MR: Mendelian Randomization
RCTs: Randomized Controlled Trials
GO: Gene Ontology
KEGG: Kyoto Encyclopedia of Genes and Genomes
GSEA: Gene Set Enrichment Analysis
DEGs: Differentially Expressed Genes
IRGs: Immune-related Genes
GWAS: Genome-wide Association Studies
TFs: Transcription Factors
GEO: Gene Expression Omnibus
SNPs: Single Nucleotide Polymorphisms
IVs: Instrumental Variables
eQTL: Expression Quantitative Trait Loci
ssGSEA: Single-sample Gene-set Enrichment Analysis
NES: Normalized Enrichment Score
AUC: Area Under the Curve
