# Supplementary material for: Identification of immune-related hub genes and potential molecular mechanisms involved in COVID-19 via integrated bioinformatics analysis
Source: Sci Rep. 2024 Dec 2;14:29964. doi: 10.1038/s41598-024-81803-2 (PMC11612211; doi:10.1038/s41598-024-81803-2)
Supplement: Supplementary file 1 — Supplementary Material 1 [file 41598_2024_81803_MOESM1_ESM.docx]

Table. 5 The enriched motifs and their corresponding transcription factors for the two key genes

| No. | Logo | GeneSet | Motif | NES | AUC | TF_highConf | nEnrGenes | EnrichedGenes |
| --- | --- | --- | --- | --- | --- | --- | --- | --- |
| 1 | 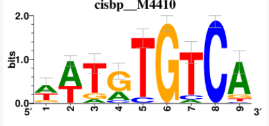 | key_gene | cisbp__M4410 | 6.18 | 0.499 | MEIS1; MEIS2; MEIS3; PKNOX1; PKNOX2; TGIF1; TGIF2; TGIF2LX; TGIF2LY (inferredBy_Orthology). | 1 | IL1B |
| 2 | 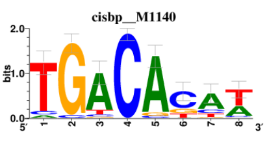 | key_gene | cisbp__M1140 | 6.16 | 0.498 | MEIS1; MEIS2; MEIS3; PKNOX1; PKNOX2; TGIF1; TGIF2; TGIF2LX; TGIF2LY (inferredBy_Orthology). | 1 | IL1B |
| 3 | 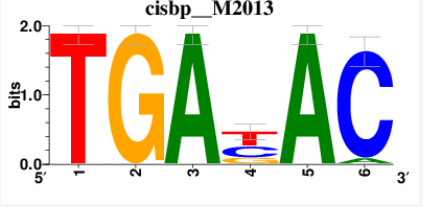 | key_gene | cisbp__M2013 | 6.16 | 0.497 | SIX4; SIX5 (inferredBy_Orthology). | 1 | IL1B |
| 4 | 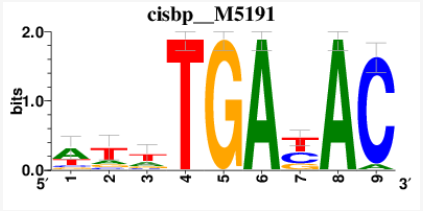 | key_gene | cisbp__M5191 | 6.16 | 0.497 | SIX4; SIX5 (inferredBy_Orthology). | 1 | IL1B |
| 5 | 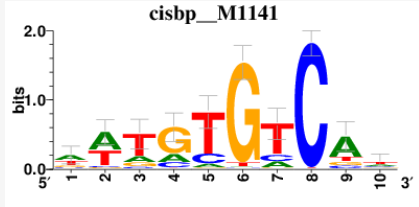 | key_gene | cisbp__M1141 | 6.14 | 0.496 | MEIS1; MEIS2; MEIS3; PKNOX1; PKNOX2; TGIF1; TGIF2; TGIF2LX; TGIF2LY (inferredBy_Orthology). | 1 | IL1B |
| 6 | 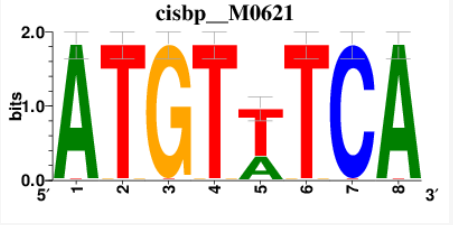 | key_gene | cisbp__M0621 | 6.09 | 0.492 |  | 2 | CD1C;IL1B |
| 7 | 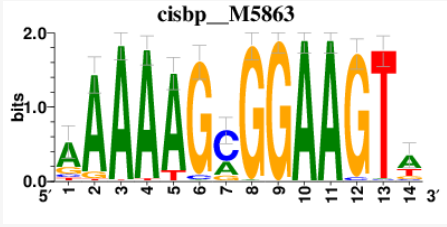 | key_gene | cisbp__M5863 | 6.08 | 0.491 | SPI1 (directAnnotation). | 1 | IL1B |
| 8 | 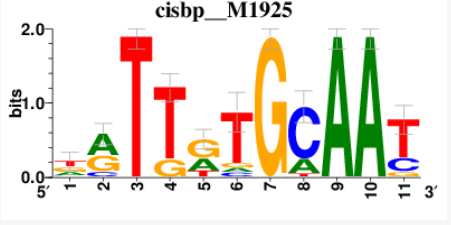 | key_gene | cisbp__M1925 | 6.06 | 0.49 | CEBPA (directAnnotation). | 1 | IL1B |
| 9 | 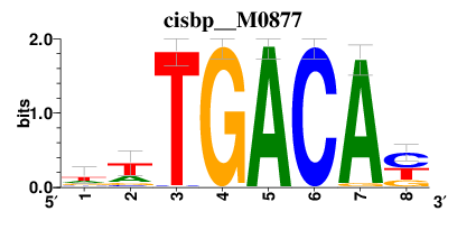 | key_gene | cisbp__M0877 | 6.05 | 0.489 |  | 1 | IL1B |
| 10 | 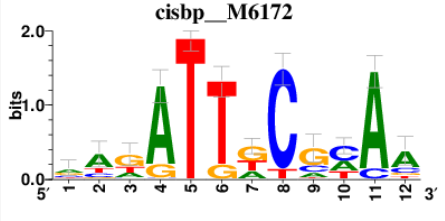 | key_gene | cisbp__M6172 | 6.03 | 0.487 | CEBPE (directAnnotation). | 1 | IL1B |
